# Compositional and functional differences in human gut microbiome with respect to equol production and its association with blood lipid level: a cross-sectional study

**DOI:** 10.1186/s13099-019-0297-6

**Published:** 2019-05-10

**Authors:** Wei Zheng, Yue Ma, Ai Zhao, Tingchao He, Na Lyu, Ziqi Pan, Geqi Mao, Yan Liu, Jing Li, Peiyu Wang, Jun Wang, Baoli Zhu, Yumei Zhang

**Affiliations:** 1 Division of Endocrinology and Metabolism, Department of Obstetrics, Beijing Obstetrics and Gynecology Hospital, Capital Medical University, Beijing, China; 2 Department of Social Medicine and Health Education, School of Public Health, Peking University Health Science Center, Beijing, China; 3 CAS Key Laboratory of Pathogenic Microbiology and Immunology, Institute of Microbiology, Chinese Academy of Science, Beichen West Road 1, Haidian District, Beijing, 100101 China; 4 University of Chinese Academy of Science, Beijing, China; 5 Beijing Key Laboratory of Microbial Drug Resistance and Resistome, Beijing, China; 6 Department of Nutrition and Food Hygiene, School of Public Health, Peking University Health Science Center, Xueyuan Road 38, Haidian District, Beijing, 100191 China; 7 Collaborative Innovation Center for Diagnosis and Treatment of Infectious Diseases, The First Affiliated Hospital, College of Medicine, Zhejiang University, Hangzhou, China; 8 Department of Pathogenic Biology, School of Basic Medical Sciences, Southwest Medical University, Zhongshan Road, Luzhou, Sichuan China; 9 Beijing Key Laboratory of Toxicological Research and Risk Assessment for Food Safety, Beijing, China

**Keywords:** Equol phenotype, Gut microbiota, Blood lipid, Soy isoflavone, Dyslipidemia

## Abstract

**Background:**

Gut microbiota affects lipid metabolism interactively with diet. Equol, a metabolite of isoflavones produced by gut bacteria, may contribute substantially in beneficial lipid-lowering effects. This study aimed to examine equol production-related gut microbiota differences among humans and its consequent association with blood lipid levels.

**Results:**

Characterization of the gut microbiota by deep shotgun sequencing and serum lipid profiles were compared between equol producers and non-producers. Gut microbiota differed significantly at the community level between equol producers and non-producers (P = 0.0062). At the individual level, 32 species associated with equol production were identified. Previously reported equol-producing related species *Adlercreutzia equolifaciens* and *Bifidobacterium bifidum* showed relatively higher abundance in this study in equol producers compared to non-producers (77.5% vs. 22.5%; 72.0% vs. 28.0%, respectively). Metabolic pathways also showed significant dissimilarity between equol producers and non-producers (P = 0.001), and seven metabolic pathways were identified to be associated with the equol concentration in urine. Previously reported equol production-related gene sequences in *A. equolifaciens* 19450T showed higher relative abundance in equol producers than in non-producers. Additionally, we found that equol production was significantly associated with the prevalence of dyslipidemia, including a marginal increase in serum lipids (27.1% vs. 50.0%, P = 0.02). Furthermore, equol production was not determined by intake of soy isoflavones, which suggested that gut microbiota is critical in the equol production process.

**Conclusion:**

Both content and functioning of the microbial gut community significantly differed between equol producers and non-producers. Further, equol producers showed lower prevalences of dyslipidemia, which suggests the important role that equol might play in lipid metabolism by gut microbiota.

**Electronic supplementary material:**

The online version of this article (10.1186/s13099-019-0297-6) contains supplementary material, which is available to authorized users.

## Background

Cardiovascular disease (CVD) is the leading cause of death globally, and dyslipidemia is a critical modifiable risk factor for its development. Recent evidence reveals that the gut microbiome is a novel target to reduce cardiometabolic risk factors such as dyslipidemia [1]. Gut microbiome exerts its effect through a complex system of microorganism–microorganism and host–microorganism interactions [2]. Emerging evidence showed that gut microbiota may regulate blood lipid metabolism independently and interactively with diet [3, 4].

Isoflavones (SI), a class of phytoestrogens that can be found in high amounts in soy foods, may play an important role in cardiometabolic health due to its antioxidant, anti-proliferative, or apoptotic effects [5–9]. It is estimated that approximately a 10% reduction in low density lipoprotein-cholesterol (LDL-C) was due to soy in comparison with animal protein [5, 10] and up to 72% of its benefit were contributed by SI [7]. However, further studies suggested that the beneficial effect of SI varied in different populations [11] may be due to its most bioactive metabolic product, equol [12]. Asians are prone to benefit from soy products compared to Western populations because approximately 50–60% of Chinese/Japanese individuals may produce equol (called an equol-producer [EP]) [13]. Equol is produced by specific colonic bacteria from its precursor daidzein, a major type of SI [12, 14], and whether individuals do or do not produce equol depends on their gut microbial community [15, 16]. Dozen strains of bacteria that are involved in equol production pathways have been isolated after considerable efforts [15, 17, 18]. However, the overall difference in gut microbiota between an EP and non-producer (NP) and its implication on blood lipid regulation remain unexamined. Development of deep shotgun sequencing and metagenome-wide association analysis enabled in-depth characterization into the content, diversity, and functioning of the microbial gut community. In our study, we determined gut microbiota using metagenome sequencing to establish a comprehensive framework of equol production-related gut microbiota differences among humans, as well as its consequent association with blood lipid levels.

## Results

### General cohort description

The present study included a total of 99 with an average age of 36 years old, including 46 males and 53 females, 59 out of which were classified to be EP according to equol excretion status in urine. A 24-h urine sample was collected after oral administration of a capsule of SI for 3 days and equol excretion was determined by high performance liquid chromatography (HPLC).

### Gut microbiota diversity in EP and NP

**Fig. 1:**
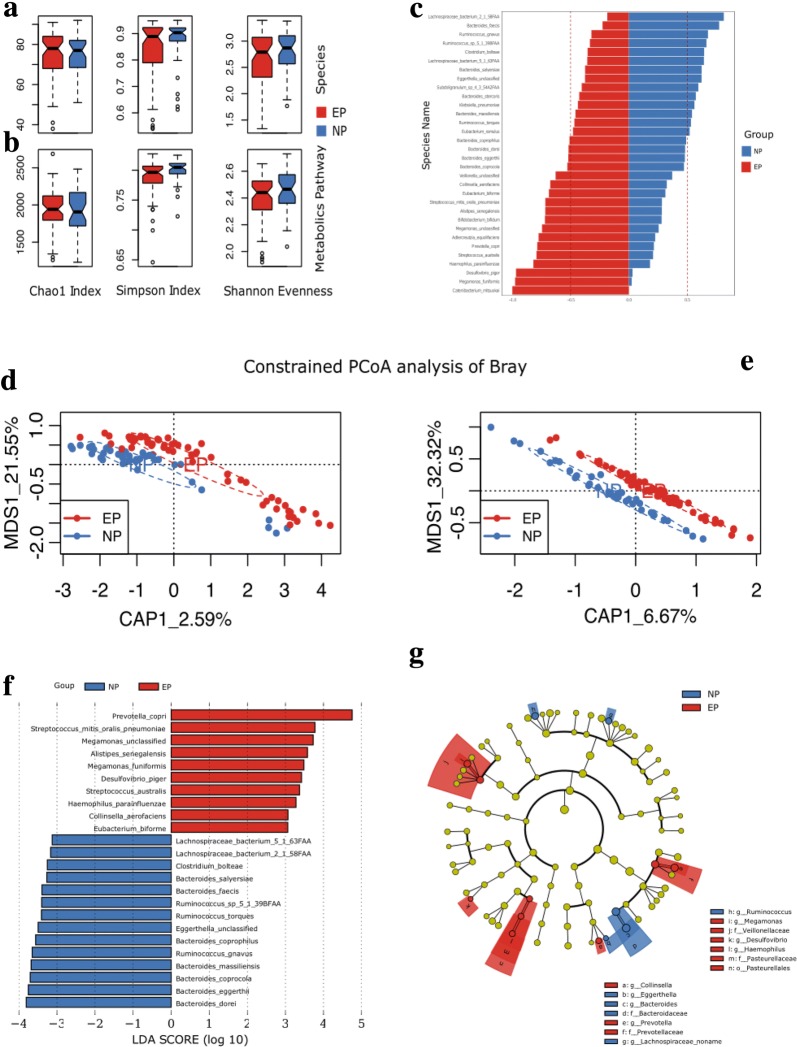
Microbiome and metagenomics diversity and LEfSe analysis. **a**, **b** Alfa-diversities of microbiome composition and functional capacities between EP (red) and NP (blue) groups. **c** Species that are significantly different in EP (red) vs. NP (blue) groups. Significance was determined using Wilcoxon rank-sum test, and the relative proportion is shown for each species. **d**, **e** Bray–Curtis based constrained principal co-ordinates analysis (PCoA) showing EP (red) and NP (blue) with significantly different taxonomical compositions and functional capacities. **f**, **g** The biomarkers identified by linear discriminant analysis effect size (LEfSe) ranked according to the effect size and associating them with the class with the highest median. The color red represents the biomarkers in the EP group and the blue color indicates biomarkers in the NP group. The length of each bar represents the linear discriminant analysis (LDA) score format with log 10

To identify the association of gut microbiome with equol phenotype, we performed shotgun metagenomics sequencing of fecal samples from study participants. The DNA sequencing data have been deposited in the BIG Data Center (accession numbers: CRA001481). Metagenomic reads from 99 fecal samples were processed with MetPhlAn2 (Metagenomic Phylogenetic Analysis) to determine composition of gut microbiome and to calculate the relative abundances of species. The microbial composition at phylum level was shown in Additional file 1: Figure S1. Phyla and species with relative abundance equal to or larger than 0.0001 of the average level in each group (EP and NP) were included in further analyses. We compared the difference in microbial composition between EP and NP adjusted for age, gender, BMI, equol phenotype and smoking habit (Table 2). Only the factor of equol was significantly associated with inter-individual microbial distance and this factor explained 2.05% of the variation in microbial composition (P = 0.02 for equol). We then discovered significant community level microbiota differences, using the Adonis test and Bray–Curtis distance matrix calculated from species-level composition between EP and NP (P = 0.0062). However, no significant difference in bacterial richness and evenness were observed between EP and NP (P = 0.64 for Shannon–Wiener index, P = 0.72 for Chao1, and P = 0.43 for Simpson index in Fig. 1a). PCoA based on the Bray–Curtis distance matrix were conducted to reveal dissimilarity in metagenome-based relative abundances between EP and NP at the species level (Fig. 1d). EP and NP are clustered into different structures of gut microbiome as indicated in Fig. 1d.

### Identified bacterial species and biomarkers associated with equol production

At individual taxa level, we used the criteria mentioned above and kept 139 from a total of 351 species for further analyses. With Wilcoxon rank-sum test, 32 species showed significant differences in two groups (Fig. 1c; P < 0.1). Equol producing-related species *Adlercreutzia equolifaciens* and *Bifidobacterium bifidum,* showed higher relative abundance in EP than in NP (77.5% vs. 22.5% and 72.0% vs. 28.0%, respectively), despite the significance being only marginal (P = 0.06 and P = 0.08). We also used LEfSe analysis for biomarker discovery within the microbiome at species level between the two groups; 14 biomarkers were defined within the NP group and 10 biomarkers within the EP group as shown in Fig. 1f, g.

### Functional diversity of the gut microbiome in EP and NP

**Fig. 2:**
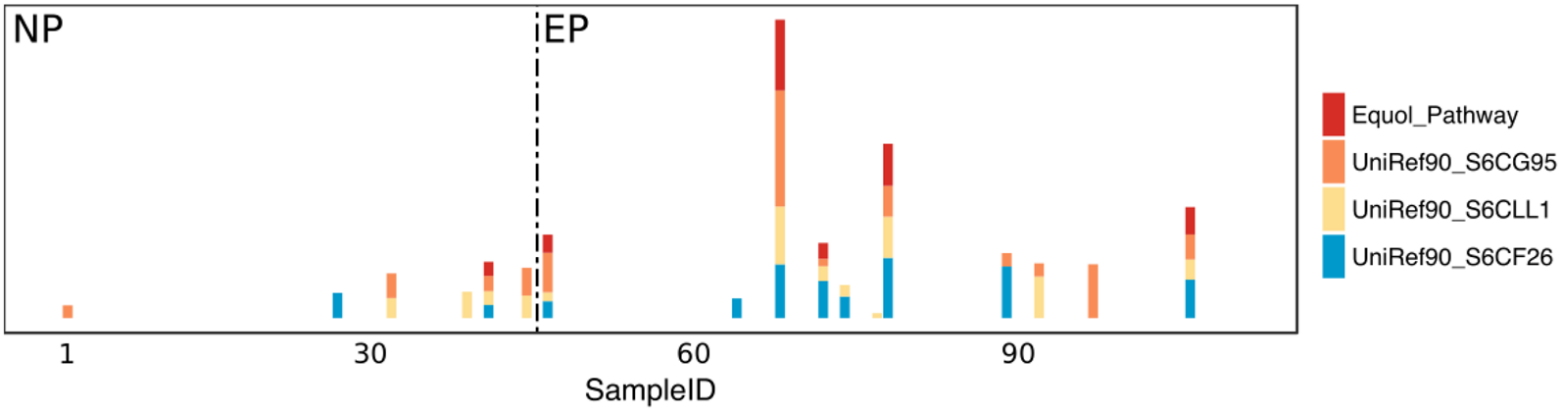
Three equol metabolic genes relative abundance and calculated equol producing pathway shows in all samples. The colors of bar represent genes and pathway exist in that sample. And the dot-line means different groups, left zone is non-producer group and right zone is equol producer group

To investigate the differences in presence/absence, as well as abundance of metabolic pathways in the gut microbial community between EP and NP, we carried out analysis on the metagenomics sequences and obtained their genomic functional potential using HUMAnN2 (the HMP Unified Metabolic Analysis Network) [19]. A total of 134 metabolic pathways with relative abundance larger than 0.0001 of the average level were included in the analysis. We identified 75 metabolic pathways, which showed significant differences (P < 0.1) between EP and NP by Wilcoxon rank-sum test, 57 of which remained statistically significant after false discovery rate (FDR) adjustment (Q < 0.1). Most of these pathways are biosynthesis pathways (55 out of 75), among which 7 pathways were significantly associated (P < 0.05) with equol concentration in urine by using Spearman correlation analysis (Additional file 1: Figure 2). Chao1 index and evenness in metabolic pathways showed no significant differences between EP and NP, and richness in metabolic pathways was higher in EP than in NP (P = 0.86 for Chao1, P = 0.12 for Shannon–Wiener index, and P = 0.015 for Simpson index in Fig. 1b). Subsequently, we conducted constrained PCoA analysis on the metabolic pathways (Fig. 1e) and statistical significance test (anova.cca) on the result of constrained PCoA, which showed significant dissimilarity between EP and NP for metabolic pathways as well (Permanova P = 0.001). We also established an equol production-related pathway based on the three critical gene sequences in *A. equolifaciens* 19450T, *Eggerthella* sp. YY7918, and *Lactococcus garvieae* to blast the reference dataset of HUMAnN2. We found only the genes from *A. equolifaciens* 19450T can be found with right annotation, and only these genes can be found in the result of HUMAnN2. So, we separated the relative abundances of these genes from the results of the gene part, then we used the relative abundances of these three genes to calculate the geometrical mean as the equol metabolic-related pathway’s abundance. As shown in Fig. 2 and Additional file 2: Table S1 and Table S2, this equol production-related pathway was mainly identified and showed higher relative abundance in EP in this study.

To assess whether the differences in abundance of metabolic pathways in the gut microbial community may explained by other characteristics of the participants, we further calculated the Bray–Curtis distance of the metabolic pathway using HUMAnN2 with gender, age, BMI, equol phenotype and smoking habit in the model. The result was similar to the composition of gut microbiome, only the equol phenotype had significant correlation to beta diversity (P = 0.0001) and explains 6.58% of the variation, while the rest of factors do not play significant roles (Table 1).

**Table 1:** Variation in microbial composition contributed by different factors

	Microbial composition		Metabolic pathway	
	R^2^	P-values	R^2^	P-values
Gender	0.01078608	0.4011	0.01145003	0.3108
Age	0.011782912	0.2761	0.01261881	0.2592
Equol phenotype	0.020533119	0.022	0.06578488	1.00E−04
BMI	0.007429074	0.7482	0.01105827	0.3365
Smoking habit	0.008483512	0.6199	0.00452271	0.8729

*BMI* body mass index

* P-value was calculated by Adonis test

### Equol phenotype and prevalence of dyslipidemia

**Fig. 3:**
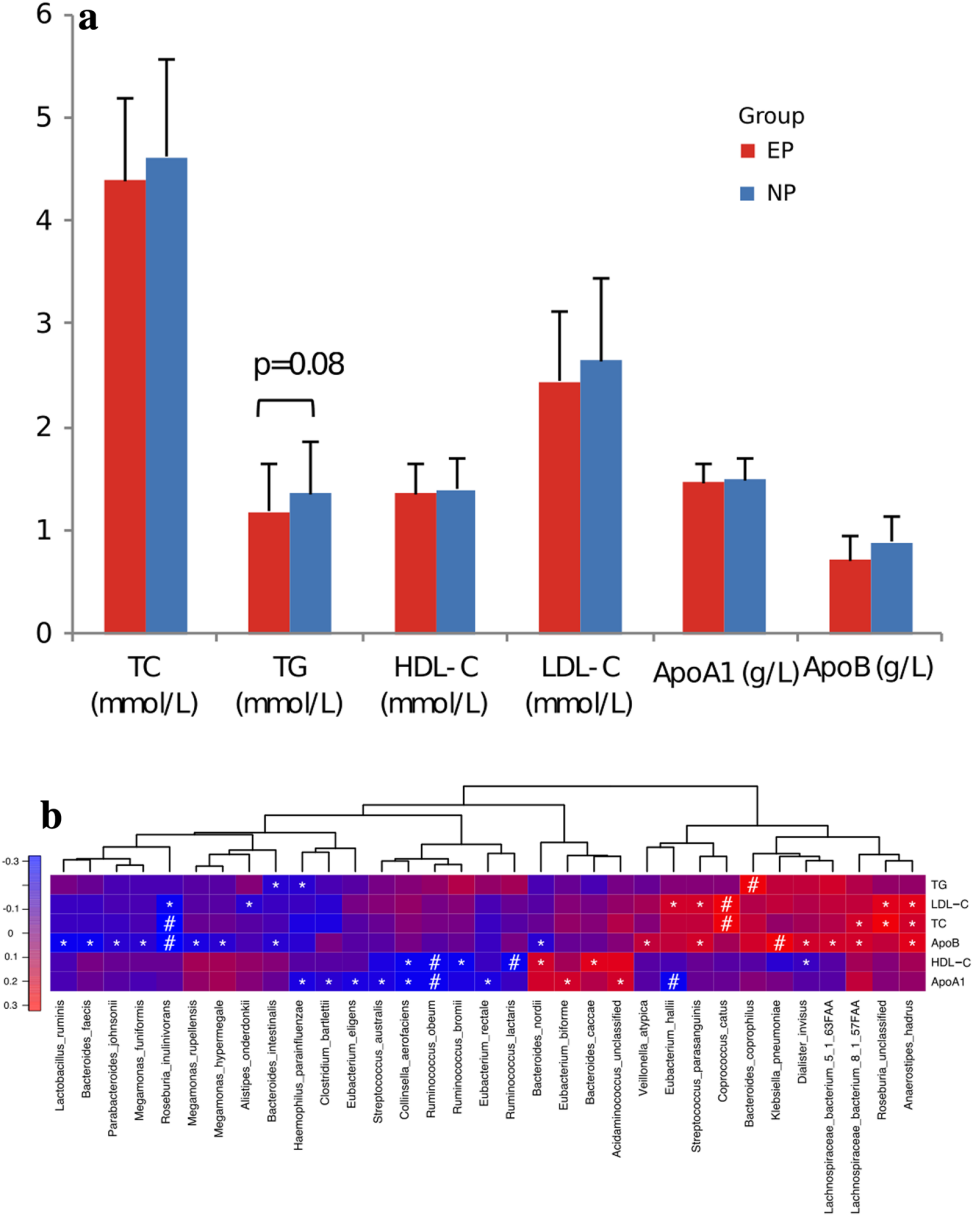
Serum lipid profiles and correlations with species. **a** Blood lipid profiles of the participants by equol phenotype (red color represent EP group and blue color represent NP group). Significant differences by t-test are indicated. **b** Correlations between species and blood lipid profiles. “*” denotes adjusted P < 0.05 and “#” denotes adjusted P < 0.01. *TG* triglycerides, *LDL-C* low density lipoprotein-cholesterol, *TC* total cholesterol, *ApoB* apolipoprotein B, *HDL-C* high density lipoprotein-cholesterol, *ApoA1* apolipoprotein A1

As shown in Table 2, a total of 36 (36.4%) of the participants were classified as having dyslipidemia. EP showed a substantially lower prevalence of dyslipidemia (27.1% vs. 50.0%, P = 0.02) than NP. Results from logistic regression models indicated that the association with dyslipidemia in serum lipid levels remained significant after adjustment for age and BMI (adjusted odds ratio = 0.37 [0.15–0.94], P = 0.036). Serum triglycerides (TG) levels was 12% lower in EP compared to NP (1.18 ± 0.48 mmol/L vs. 1.36 ± 0.57 mmol/L, P = 0.08) (Fig. 3a), although the difference between the two groups were marginally significant due to a relatively small sample size.

**Table 2:** Association between equol phenotype and classified serum lipid level

	Serum lipid level		OR^a^	Adjusted-OR^b^
	Normal	Dyslipidemia		
Equol phenotype, n (%)				
EP	43 (72.9)	16 (27.2)	0.30(0.13–0.68)	0.36(0.15–0.87)
NP	20 (50.0)	20 (50.0)	Ref.	Ref.

*EP* equol producer, *NP* non-producer

^a^Odds ratio (OR) indicated OR of being dyslipidemia calculated by logistic regression model

^b^Adjusted-OR was adjusted for age and BMI

We also tested the correlations between bacterial species and serum lipid levels from our dataset and found several species with FDR < 0.05. Negative association of *Haemophilus parainfluenzae* and positive association of *Klebsiella pneumoniae* and *Lachnospiraceae bacterium*-8_1_57FAA with total cholesterol (TC), LDL-C, and apolipoprotein B (ApoB) were observed (Fig. 3b). As is indicated in Fig. 1c, EP shows a higher relative abundance of *H. parainfluenzae* and lower levels of *K. pneumoniae* and *L. bacterium*-8_1_57FAA compared to NP. The findings suggest that these species might contribute to the changes in blood lipid levels via equol production.

### Equol concentration in urine is determined by microbiota and not food intake of soy isoflavones

Intake of SI was calculated according to a 3-day, 24-h dietary record. We first compared intake of SI (including total SI, daidzein, genistein, and glycitein) and soy products (including tofu, dried bean curd, soy bean milk, soy milk, dried beans, fermented bean curd, fermented soy beans, and other soy products) between EP and NP. No significant differences between the two groups were observed (Additional file 2: Tables S3, S4). Subsequently, we examined the correlation between intake of daidzein (the precursor to equol) and equol concentration in urine/equol excretion in 24 h in EP by Pearson correlation analysis. The results showed that neither equol concentration nor equol excretion in 24 h was associated with the amount of intake of the precursor, daidzein (r = 0.12, P = 0.3 and r = 0.07, P = 0.5, respectively). These findings suggest that equol production is mostly determined by the gut microbiome of the participants, while the intake of soy isoflavones plays a minimal role if any.

## Discussion

In this study, we found that equol production from daidzein is associated with differences in gut microbiome in humans. We observed, both in terms of composition and functional capacities, EPs contain a distinct microbial profile in their gastrointestinal tract compared to that of NPs. We identified a list of bacteria with equol producing potential in the comparative analysis including *A. equolifaciens* and *B. bifidum,* which have been tested to show equol production ability in vitro by Maruo et al. [20] and Raimondi et al. [17], respectively. However, the exact role of these species remains to be examined. In terms of functional capacity, we compared metabolic pathways in EP and NP using HUMAnN2, and found no labeled equol production-related metabolic pathways. But this was mainly due to a misclassification of the analytical method, as when we used the three previously reported gene sequences (UniRef90-S6CF26, UniRef90-S6CLL1, and UniRef90-S6CG95) pathways in the results, those pathways indeed also showed a higher relative abundance in EP.

The role of microbiome in shaping equol production has crucial health implications. As the most bioactive metabolite of SI, equol becomes an area of research focus. This study indicated that EP had a remarkably lower prevalence of dyslipidemia, which were consistent with previous reports [12]. Cardiovascular benefit of maintaining elevated high density lipoprotein-cholesterol (HDL-C) levels has been observed in EP in epidemiological studies [21]. Thus, equol production-related bacteria might also affect human health. In a previous study, Fu et al. [1] showed that human gut microbiomes are associated with overall blood lipid levels, and the composition of gut microbiomes are involved in the development of CVD through different blood lipids (HDL-C and TG). Our study further provides one of the probable mechanistic explanations for the microbiome association with blood lipid levels, with equol acting as an intermediary. For instance, we found the genus *Eggerthella,* a genus identified in Fu et al. [1], tends to be more abundant within the NP group, showing positive correlation with TG and negative correlation with HDL-C; the trends in these two lipids are at the same time associated with a low risk of CVD. This study also identified several additional bacterial species associated with equol phenotype and lipid profiles.

A recent review by Frankenfeld et al. [9] indicated that gut microbiota–phytoestrogen (especially SI) interactions may serve as a novel target for reducing cardiometabolic risk. The equol phenotype present widely influenced cardiovascular, bone, and menopausal health, as well as hormone-related cancers such as breast cancer and prostate cancer [12, 22]. Therefore, to characterize gut microbiota of EP and NP by deep shotgun sequencing indicates important health implications, such as risk assessment and management in a variety of diseases.

This study has certain limitations. We could not make causal inferences in a population-based cross-sectional study. More experiments are needed to examine the effect of gut microbiota on isoflavone metabolism, blood lipid regulation, and, eventually, health benefits.

## Conclusion

In summary, our study discovered compositional and functional differences in human gut microbiome with regard to equol production; previously reported members of the gut microbial communities and pathways also showed differences between EP and NP. These differences could have important impacts on human blood lipid levels and related health status.

## Methods

### Participants

This study recruited adults aged 18–65 years in Beijing, China. Individuals with digestive system diseases; infectious diseases or diabetes; who used antibiotics, intestine and stomach drugs, and hormonal drugs during the past month; and women who were pregnant or lactating were excluded. The volunteers who met the above conditions were tested for fasting blood glucose (FBG). Those with a FBG ≥ 6.1 mmol/L were excluded. This study was approved by the Ethical Committee of the Health Science Center at Peking University (NO. IRB00001052-15046). Written informed consent forms were collected from the participants.

### SI intake assessment and anthropometric measurements

Information on dietary intake was collected by a 3-day, 24-h dietary record and food frequency questionnaire. SI and other nutrient intakes were calculated according to China Food Composition, 2009 [23]. Standard Tables of Food Composition in Japan 2010 [24] was used as a supplementary standard in case the food intake was not included in China Food Composition, 2009. Anthropometric measurements were carried out by trained investigators using a standardized protocol. Body mass index (BMI) was calculated as weight/height^2^ (kg/m^2^).

### Equol phenotype determination

According to previous reports, soy extract isoflavone challenging increased urinary equol excretion; and, thus, helpful to assess the potential ability of equol production [13]. Therefore, in this study, we determined equol phenotype after a 3 day isoflavone challenge. Each participant was orally administrated one capsule of SI (North China Pharmaceutical Group Co., Ltd, Hebei, China) for three consecutive days. The capsule contained 22.6 mg daidzin, 0.38 mg daidzein, 1.07 mg genistin, 0.32 mg genistein, 1.75 mg glycitin, and 0.18 mg glycitein. From the morning of the third day, each participant voided their bladder and began to collect a complete pooled 24-h urine sample.

S-Equol, daidzein, genistein, and glycitein concentrations were determined by HPLC [13]. The column was Capcell PACK UG120 5 μm 4.6 φ × 250 mm (Shiseido Co., Ltd., Japan). The quantification of the SI was achieved by calculating the area ratio of the SI to its stable-labeled analog and interpolation of the value against calibration curves constructed of known concentrations of pure standards. Those with positive equol excretion in urine were classified to be EP.

### Blood lipid profiles determination

Venous blood samples were collected following an overnight fast. TC, TG, and HDL-C were assayed by enzymatic methods using an autoanalyzer (Modular P-800; Roche, Switzerland). Concentration of LDL-C was calculated from the Friedewald equation (LDL-C = TC − (HDL-C + TG/5)). Dyslipidemia was defined according to Guidelines for Prevention and Treatment of Dyslipidemia in Chinese Adults (revised in 2016) [25].

### Stool sample collection, DNA extraction, and sequencing

Stool samples were collected on the fourth day after the participants took one capsule of SI for three consecutive days using a stool store kit (PSP^®^ Spin Stool DNA Plus Kit) with preservation solution. DNA was extracted from stool using the TIANamp Stool DNA Kit as described by Manichanh et al. [26]. We conducted quality control using nanodrop instrument and agarose gel electrophoresis. Metagenomics library was constructed by NEXTflex Rapid DNA-Seq Kit (Illumina). The procedures included cluster generation, template hybridization, isothermal amplification, linearization, blocking and denaturation, and hybridization of the sequencing primers. The primers used in this process are PP1 (AATGATACGGCGACCACCGAGATCTACAC) and PP2 (CAAGCAGAAGACGGCATACGAGAT). We constructed paired-end metagenomics library with 450 bp insert size for each samples, sequencing on Illumina HiSeq 2500 platform, and obtained around 3 million paired-end reads for each samples.

### Metagenomics analysis

To remove low quality sequence reads, we used SoapAligner [27] (version 2.21) with default parameters. Human contamination was removed by using bowtie2 (version 2.2.6). To predict the composition of microbiota, we used software MetaPhlan2 [19] (version 2.6.0) with default parameters. The reference set of MetaPhlan2 contains about 1 million unique markers genes from 17,000 species (13,500 bacterial and archaeal, 3500 viral, and 110 eukaryotic). The profile of microbial function was constructed using HUMAnN2 [28] (version 0.11.1) with default pipeline parameters. The process of HUMAnN2 relied on the reference from the UniPort Reference Clusters (UniRef50). Subsequently, we collected and downloaded three genes from previously reported equol metabolic pathways and used local blast (version 2.2.28) software to map the three genes with the UniRef50 protein dataset as the reference dataset. Biomarkers within the microbiome at the species level were explored using linear discriminant analysis effect size (LEfSe) [29].

### Statistical analysis

#### Diversity of microbiota and metagenomics

We measured the feature of microbial composition using three different alpha diversity indices, two of which (Shannon–Wiener and Simpson) were calculated using the function *diversity* in R package vegan (version 2.4-4), and Chao1’s diversity index was calculated using the function *estimateR* from the same R package. We chose the Shannon–Wiener index and Simpson index to calculate the microbial function alpha diversity. Subsequently, we calculated the Bray–Curtis distance of the above two indices with their relative abundance dataset to examine the beta diversity of microbial composition and function using function of *vegdist* in R package vegan. Then, we conducted constrained principal coordinate analysis (constrained PCoA) using the function of *capscale* in the same package.

#### Wilcoxon rank-sum test

The differential in abundance of species, pathways, and all five alpha diversity indices were tested by two-tailed Wilcoxon rank-sum test using function *wilox.test* from R package stats (version 3.4.1). To control the false discovery rate, we used function *p.adjust* to adjust the P values at the last step (P value correction method was Benjamini and Hochberg method or its alias FDR). The threshold of transformed Q values was defined to be 0.1.

#### Analysis of variance and permutational multivariate analysis of variance

We compared the difference in microbial composition and function distance matrix between EP and NP using the function *adonis* in the R package vegan. We determined how variations of species’ Bray–Curtis distances were explained by characteristics of the participants. Four factors including age, gender, BMI, and equol phenotype were included in the model. Meanwhile, we used the function *anova* in R package stats to test the significance of the result from PCoA. In all of these, the P value was determined by 10,000× permutations and the threshold was 0.05.

#### Association between equol phenotype, related species, and serum lipid levels

The association between equol phenotype and prevalence of dyslipidemia were examined by multivariate logistic regression model adjusted for age and BMI. To assess the effect of gut microbiome on serum lipid levels, the Spearman correlation coefficients between lipid profiles and relative abundance in species were calculated using the function *corr.test* in R package psych (version 1.7.8). The P values were adjusted for using the Benjamini and Hochberg method.

#### Association between equol production rate and soy food intake

To examine whether equol production rate was associated with intake of soy food or SI, we examined the association between equol production/concentration in urine and food intake using the Spearman correlation and Chi-square test. The P values for Spearman correlation were adjusted using the Benjamini and Hochberg method.

## Additional files

**Additional file 1: Figure S1.** The microbial composition of each individual is shown at phylum level. The individuals are sorted by the abundance of Bacteroidetes. **Figure S2.** Metabolic pathways associated with equol concentration in urine. “*” denotes P < 0.05 and “#” denotes P < 0.01.

**Additional file 2: Table S1.** Comparison of relative abundance of production-related pathways in EP and NP. **Table S2.** Diversity in metabolic pathways between NP and EP. **Table S3.** Estimated intake of soy bean products in the participants by equol phenotype according to Food Frequency Questionnaire survey. **Table S4.** Estimated intake of SI in the participants by equol phenotype according to 24 h diet records during past 3 days (mg/day).

## Abbreviations

EP: equol producer
NP: non-producer
SI: soy isoflavone
LDL-C: low density lipoprotein-cholesterol
FBG: fasting blood glucose
BMI: body mass index
HPLC: high performance liquid chromatography
TC: total cholesterol
TG: triglycerides
HDL-C: high density lipoprotein-cholesterol
ApoA1: apolipoprotein A1
ApoB: apolipoprotein B
PCoA: principal co-ordinates analysis
LEfSe: linear discriminant analysis effect size
LDA: linear discriminant analysis
