# Supplementary material for: Compositional and functional differences in human gut microbiome with respect to equol production and its association with blood lipid level: a cross-sectional study
Source: Gut Pathog. 2019 May 10;11:20. doi: 10.1186/s13099-019-0297-6 (PMC6509798; doi:10.1186/s13099-019-0297-6)
Supplement: Supplementary file 1 — Additional file 1: Figure S1. The microbial composition of each individual is shown at phylum level. The individuals are sorted by the abundance of Bacteroidetes. Figure S2. Metabolic pathways associated with equol concentration in urine. “*” denotes P < 0.05 and “#” denotes P < 0.01. [file 13099_2019_297_MOESM1_ESM.pptx]

## Slide 1
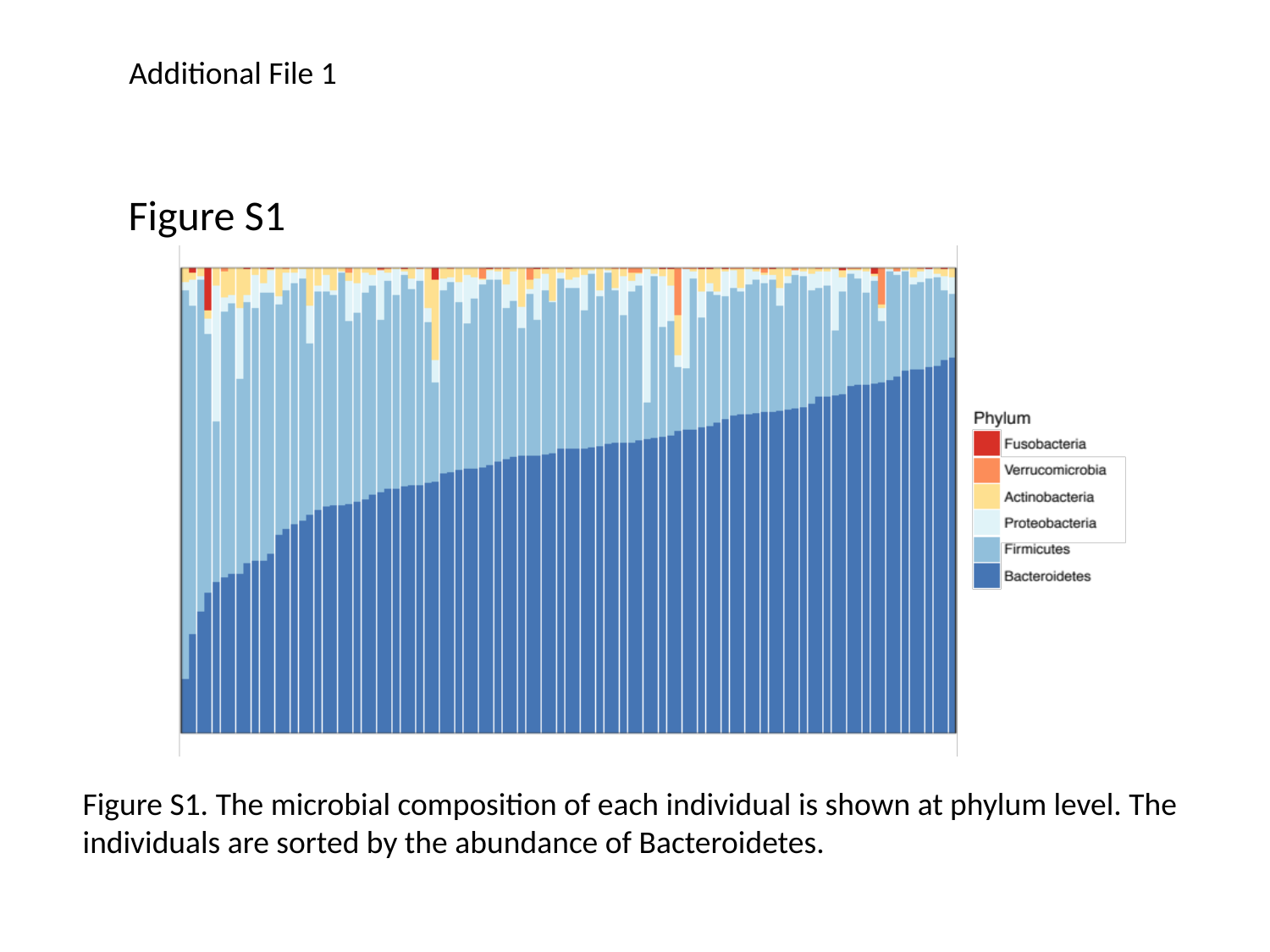

Additional File 1
Figure S1
Figure S1. The microbial composition of each individual is shown at phylum level. The individuals are sorted by the abundance of Bacteroidetes.

## Slide 2
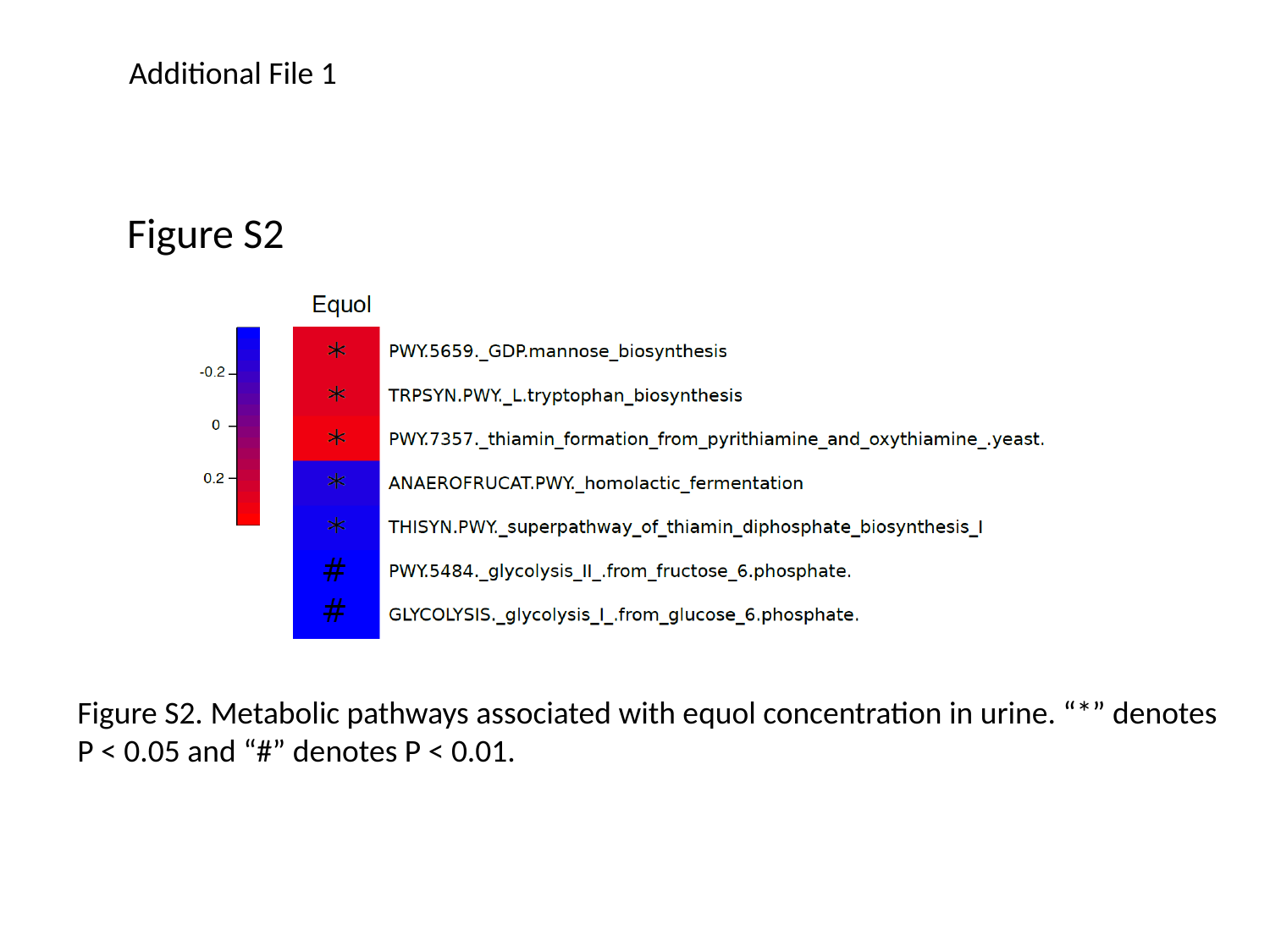

Additional File 1
Figure S2
Figure S2. Metabolic pathways associated with equol concentration in urine. “*” denotes P < 0.05 and “#” denotes P < 0.01.
