# Supplementary material for: Compositional and functional differences in human gut microbiome with respect to equol production and its association with blood lipid level: a cross-sectional study
Source: Gut Pathog. 2019 May 10;11:20. doi: 10.1186/s13099-019-0297-6 (PMC6509798; doi:10.1186/s13099-019-0297-6)
Supplement: Supplementary file 2 — Additional file 2: Table S1. Comparison of relative abundance of production-related pathways in EP and NP. Table S2. Diversity in metabolic pathways between NP and EP. Table S3. Estimated intake of soy bean products in the participants by equol phenotype according to Food Frequency Questionnaire survey. Table S4. Estimated intake of SI in the participants by equol phenotype according to 24 h diet records during past 3 days (mg/day). [file 13099_2019_297_MOESM2_ESM.docx]

Additional File 2

Table S1. Comparison of relative abundance of production-related pathways in EP and NP

|  | **EP** | **NP** |
| --- | --- | --- |
| **UniRef90_S6CG95** | 2.494E-07 | 9.286E-07 |
| **UniRef90_S6CLL1** | 2.564E-07 | 6.222E-07 |
| **UniRef90_S6CF26** | 1.186E-07 | 9.277E-07 |
| **Equol_Pathway** | 4.36E-08 | 5.401E-07 |

EP, equol producer; NP, non-producer.

Additional File 2

Table S2. Diversity in metabolic pathways between NP and EP

| Pathway | NP | EP | p.values | p.adjust |
| --- | --- | --- | --- | --- |
| PWY-6387:_UDP-N-acetylmuramoyl-pentapeptide_biosynthesis_I_(meso-diaminopimelate_containing) | **0.00051911** | **0.00062686** | **2.6274E-05** | **0.00149423** |
| PYRIDNUCSYN-PWY:_NAD_biosynthesis_I_(from_aspartate) | **9.9911E-05** | **0.00016238** | **2.7113E-05** | **0.00149423** |
| PWY-6386:_UDP-N-acetylmuramoyl-pentapeptide_biosynthesis_II_(lysine-containing) | **0.00053961** | **0.0006478** | **3.2721E-05** | **0.00149423** |
| PEPTIDOGLYCANSYN-PWY:_peptidoglycan_biosynthesis_I_(meso-diaminopimelate_containing) | **0.00052392** | **0.00063088** | **5.1946E-05** | **0.00177914** |
| DTDPRHAMSYN-PWY:_dTDP-L-rhamnose_biosynthesis_I\|unclassified | **8.9636E-05** | **0.00015019** | **0.00013426** | **0.00306559** |
| PWY-7219:_adenosine_ribonucleotides_de_novo_biosynthesis | **0.00074684** | **0.00090075** | **0.00017882** | **0.0034997** |
| COMPLETE-ARO-PWY:_superpathway_of_aromatic_amino_acid_biosynthesis | **0.00041583** | **0.00048334** | **0.00028002** | **0.00438196** |
| NONMEVIPP-PWY:_methylerythritol_phosphate_pathway_I | **0.00051123** | **0.00061219** | **0.00041044** | **0.00507096** |
| COMPLETE-ARO-PWY:_superpathway_of_aromatic_amino_acid_biosynthesis\|unclassified | **9.6235E-05** | **0.00015018** | **0.00052217** | **0.00507096** |
| ARO-PWY:_chorismate_biosynthesis_I | **0.00041607** | **0.00048143** | **0.0005222** | **0.00507096** |
| ARO-PWY:_chorismate_biosynthesis_I\|unclassified | **9.9398E-05** | **0.00015299** | **0.00062808** | **0.00507096** |
| PWY-6386:_UDP-N-acetylmuramoyl-pentapeptide_biosynthesis_II_(lysine-containing)\|unclassified | **0.00011464** | **0.00018081** | **0.00071559** | **0.00507096** |
| PWY-5188:_tetrapyrrole_biosynthesis_I_(from_glutamate) | **8.9031E-05** | **0.00011322** | **0.00074389** | **0.00507096** |
| COA-PWY-1:_coenzyme_A_biosynthesis_II_(mammalian)\|unclassified | **9.1223E-05** | **0.00013764** | **0.00074394** | **0.00507096** |
| NONMEVIPP-PWY:_methylerythritol_phosphate_pathway_I\|unclassified | **0.00010571** | **0.00017178** | **0.00075363** | **0.00507096** |
| PWY-5686:_UMP_biosynthesis | **0.00055258** | **0.00065223** | **0.00075366** | **0.00507096** |
| PWY-6163:_chorismate_biosynthesis_from_3-dehydroquinate\|unclassified | **0.00010215** | **0.00016381** | **0.0007734** | **0.00507096** |
| BRANCHED-CHAIN-AA-SYN-PWY:_superpathway_of_branched_amino_acid_biosynthesis | **0.00024545** | **0.00030424** | **0.00078342** | **0.00507096** |
| PWY-7221:_guanosine_ribonucleotides_de_novo_biosynthesis\|unclassified | **0.00010843** | **0.00016697** | **0.00081432** | **0.00507096** |
| PWY-7221:_guanosine_ribonucleotides_de_novo_biosynthesis | **0.0005531** | **0.000669** | **0.00087947** | **0.00523859** |
| PWY-6387:_UDP-N-acetylmuramoyl-pentapeptide_biosynthesis_I_(meso-diaminopimelate_containing)\|unclassified | **0.0001105** | **0.00017408** | **0.0009494** | **0.00541947** |
| PWY-5097:_L-lysine_biosynthesis_VI\|unclassified | **8.3921E-05** | **0.00013273** | **0.00101152** | **0.00554315** |
| TRNA-CHARGING-PWY:_tRNA_charging | **0.00026078** | **0.00034096** | **0.00107759** | **0.00567807** |
| PWY-5103:_L-isoleucine_biosynthesis_III | **0.00021335** | **0.00026691** | **0.00125244** | **0.00614129** |
| PWY0-1319:_CDP-diacylglycerol_biosynthesis_II\|unclassified | **8.0009E-05** | **0.00012613** | **0.00129998** | **0.00614129** |
| PWY-5667:_CDP-diacylglycerol_biosynthesis_I\|unclassified | **8.0009E-05** | **0.00012613** | **0.00129998** | **0.00614129** |
| PWY-6163:_chorismate_biosynthesis_from_3-dehydroquinate | **0.00043462** | **0.00050573** | **0.00148938** | **0.0065821** |
| PWY-6737:_starch_degradation_V\|unclassified | **0.00012564** | **0.00017877** | **0.00148938** | **0.0065821** |
| PEPTIDOGLYCANSYN-PWY:_peptidoglycan_biosynthesis_I_(meso-diaminopimelate_containing)\|unclassified | **9.8597E-05** | **0.00015831** | **0.00166276** | **0.00711871** |
| PWY-7219:_adenosine_ribonucleotides_de_novo_biosynthesis\|unclassified | **0.00016081** | **0.00024865** | **0.00181036** | **0.00751574** |
| PWY-2942:_L-lysine_biosynthesis_III | **0.00049269** | **0.00059749** | **0.00271333** | **0.01032574** |
| PWY-5097:_L-lysine_biosynthesis_VI | **0.00051868** | **0.00062544** | **0.00271333** | **0.01032574** |
| PWY-6151:_S-adenosyl-L-methionine_cycle_I\|unclassified | **0.00010311** | **0.00016392** | **0.00271333** | **0.01032574** |
| PWY-6700:_queuosine_biosynthesis\|unclassified | **8.3586E-05** | **0.00014294** | **0.00301275** | **0.01115532** |
| PWY-7187:_pyrimidine_deoxyribonucleotides_de_novo_biosynthesis_II | **0.00013889** | **0.00016446** | **0.003267** | **0.0117784** |
| TRNA-CHARGING-PWY:_tRNA_charging\|unclassified | **0.00010792** | **0.00015934** | **0.00383378** | **0.01346739** |
| ILEUSYN-PWY:_L-isoleucine_biosynthesis_I_(from_threonine)\|unclassified | **9.6907E-05** | **0.00013903** | **0.00405686** | **0.01355586** |
| VALSYN-PWY:_L-valine_biosynthesis\|unclassified | **9.6907E-05** | **0.00013903** | **0.00405686** | **0.01355586** |
| PWY-7111:_pyruvate_fermentation_to_isobutanol_(engineered)\|unclassified | **9.6776E-05** | **0.00013843** | **0.00485259** | **0.01582868** |
| PWY-5686:_UMP_biosynthesis\|unclassified | **0.00011355** | **0.00015289** | **0.00617741** | **0.01968152** |
| PWY-6122:_5-aminoimidazole_ribonucleotide_biosynthesis_II\|unclassified | **0.00010681** | **0.00014288** | **0.00659124** | **0.02006665** |
| PWY-6277:_superpathway_of_5-aminoimidazole_ribonucleotide_biosynthesis\|unclassified | **0.00010681** | **0.00014288** | **0.00659124** | **0.02006665** |
| PWY-6122:_5-aminoimidazole_ribonucleotide_biosynthesis_II | **0.00034694** | **0.00040073** | **0.00954131** | **0.02751538** |
| PWY-6277:_superpathway_of_5-aminoimidazole_ribonucleotide_biosynthesis | **0.00034694** | **0.00040073** | **0.00954131** | **0.02751538** |
| PWY0-1296:_purine_ribonucleosides_degradation\|unclassified | **8.2122E-05** | **0.00011917** | **0.00964043** | **0.02751538** |
| PWY-724:_superpathway_of_L-lysine,_L-threonine_and_L-methionine_biosynthesis_II | **0.00030587** | **0.0003476** | **0.00994423** | **0.02780325** |
| PWY-6121:_5-aminoimidazole_ribonucleotide_biosynthesis_I\|unclassified | **0.00010157** | **0.0001341** | **0.01015132** | **0.02781461** |
| PWY-3001:_superpathway_of_L-isoleucine_biosynthesis_I | **0.00021063** | **0.00024457** | **0.01057705** | **0.02841285** |
| TRPSYN-PWY:_L-tryptophan_biosynthesis | **0.00012603** | **0.00016088** | **0.01195107** | **0.03148646** |
| PWY-6168:_flavin_biosynthesis_III_(fungi) | **0.00020333** | **0.00016923** | **0.01219485** | **0.03152254** |
| PWY-6737:_starch_degradation_V | **0.00048431** | **0.00056022** | **0.01321462** | **0.03352599** |
| PYRIDOXSYN-PWY:_pyridoxal_5'-phosphate_biosynthesis_I | **0.00017974** | **0.00014089** | **0.01518163** | **0.03781606** |
| PWY66-422:_D-galactose_degradation_V_(Leloir_pathway) | **0.00020832** | **0.00024013** | **0.01879466** | **0.04597979** |
| PWY-6317:_galactose_degradation_I_(Leloir_pathway) | **0.00020818** | **0.00023991** | **0.02271041** | **0.05458466** |
| PWY-7357:_thiamin_formation_from_pyrithiamine_and_oxythiamine_(yeast) | **0.00023143** | **0.00027261** | **0.03156169** | **0.07455088** |
| PWY-6897:_thiamin_salvage_II | **0.00024435** | **0.00028558** | **0.03213017** | **0.07460734** |
| ARGININE-SYN4-PWY:_L-ornithine_de_novo__biosynthesis | **0.00016471** | **0.00013393** | **0.03898461** | **0.08901485** |
| PWY66-400:_glycolysis_VI_(metazoan) | **0.00010176** | **0.00013022** | **0.04586719** | **0.1030132** |
| DENOVOPURINE2-PWY:_superpathway_of_purine_nucleotides_de_novo_biosynthesis_II | **0.00015586** | **0.00017242** | **0.04704319** | **0.10395027** |
| PWY-6527:_stachyose_degradation | **0.00017524** | **0.00020131** | **0.05463917** | **0.11881852** |
| PRPP-PWY:_superpathway_of_histidine,_purine,_and_pyrimidine_biosynthesis | **0.00010681** | **0.00011732** | **0.05645707** | **0.12085342** |
| PWY0-845:_superpathway_of_pyridoxal_5'-phosphate_biosynthesis_and_salvage | **0.00018198** | **0.00015192** | **0.06222507** | **0.13115129** |
| PWY-6151:_S-adenosyl-L-methionine_cycle_I | **0.00057669** | **0.00065451** | **0.06323183** | **0.13125395** |
| PWY-6121:_5-aminoimidazole_ribonucleotide_biosynthesis_I | **0.0003652** | **0.00040494** | **0.06425201** | **0.13138098** |
| CALVIN-PWY:_Calvin-Benson-Bassham_cycle | **0.00031944** | **0.00034868** | **0.07177984** | **0.14461527** |
| GLYCOGENSYNTH-PWY:_glycogen_biosynthesis_I_(from_ADP-D-Glucose) | **0.00011625** | **0.00013715** | **0.0794018** | **0.15016588** |
| DTDPRHAMSYN-PWY:_dTDP-L-rhamnose_biosynthesis_I | **0.00027497** | **0.00032416** | **0.0800154** | **0.15016588** |
| ILEUSYN-PWY:_L-isoleucine_biosynthesis_I_(from_threonine) | **0.00048538** | **0.00052853** | **0.0800154** | **0.15016588** |
| PWY-7111:_pyruvate_fermentation_to_isobutanol_(engineered) | **0.00048538** | **0.00052853** | **0.0800154** | **0.15016588** |
| VALSYN-PWY:_L-valine_biosynthesis | **0.00048538** | **0.00052853** | **0.0800154** | **0.15016588** |
| GALACT-GLUCUROCAT-PWY:_superpathway_of_hexuronide_and_hexuronate_degradation | **9.5035E-05** | **0.00010813** | **0.08700653** | **0.16014601** |
| PWY0-1296:_purine_ribonucleosides_degradation | **0.00027159** | **0.00030222** | **0.08767117** | **0.16014601** |
| PWY-6703:_preQ0_biosynthesis | **0.00038846** | **0.0003696** | **0.09309567** | **0.16781719** |
| PWY-6700:_queuosine_biosynthesis | **0.00055188** | **0.00063043** | **0.0959072** | **0.17064008** |
| 1CMET2-PWY:_N10-formyl-tetrahydrofolate_biosynthesis | **0.00035776** | **0.00032593** | **0.09878629** | **0.17350925** |

^*^p-value was calculated by Wilcoxon rank-sum test.

EP, equol producer; NP, non-producer.

Additional File 2

Table S3. Estimated intake of soy bean products in the participants by equol phenotype according to Food Frequency Questionnaire survey

|  | **EP** | **NP** | **p-value^*^** |
| --- | --- | --- | --- |
| **Tofu** |  |  | 0.4 |
| **Yes** | 54 (91.5) | 38 (86.4) |  |
| **No** | 5 (8.5) | 6 (13.6) |  |
| **Average amount in consumers (g/d)** | 29.1±26.5 | 33.4±45.9 | 1 |
| **Dried bean curd** |  |  | 0.6 |
| **Yes** | 33 (55.0) | 22 (50.0) |  |
| **No** | 27 (45.0) | 22 (50.0) |  |
| **Average amount in consumers (g/d)** | 15.6±21.4 | 12.4±12.4 |  |
| **Soy bean milk** |  |  | 0.6 |
| **Yes** | 32 (53.3) | 21 (47.7) |  |
| **No** | 28 (46.7) | 23 (52.3) |  |
| **Average amount in consumers (ml/d)** | 91.0±73.9 | 108.9±113.4 |  |
| **Soy milk** |  |  | 0.8 |
| **Yes** | 2 (3.3) | 2 (4.6) |  |
| **No** | 58 (96.7) | 42 (95.5) |  |
| **Average amount in consumers (ml/d)** | 29.1±26.5 | 33.4±45.9 |  |
| **Dried beans** |  |  | 0.5 |
| **Yes** | 27 (45.0) | 17 (38.6) |  |
| **No** | 33 (55.0) | 27 (61.4) |  |
| **Average amount in consumers (g/d)** | 8.2±6.1 | 8.7±10.5 |  |
| **Fermented bean curd** |  |  | 0.5 |
| **Yes** | 14 (23.3) | 13 (29.6) |  |
| **No** | 46 (76.7) | 31 (70.5) |  |
| **Average amount in consumers (g/d)** | 3.3±3.3 | 1.4±1.2 |  |
| **Fermented soy beans** |  |  | 0.2 |
| **Yes** | 16 (26.7) | 7 (15.9) |  |
| **No** | 44 (73.3) | 37 (84.1) |  |
| **Average amount in consumers (g/d)** | 28.2±77.5 | 2.4±2.7 |  |

^*^p-value was calculated by χ² test.

EP, equol producer; NP, non-producer.

Additional File 2

Table S4. Estimated intake of SI in the participants by equol phenotype according to 24h diet records during past 3d (mg/d)

|  | **EP** | **NP** | **p-value^*^** |
| --- | --- | --- | --- |
| **Total SI** | 20.6±22.2 | 20.9±34.3 | 1 |
| **Dai** | 8.6±9.4 | 8.9±15.2 | 0.9 |
| **Gen** | 11.3±12 | 11.1±17.2 | 1 |
| **Gly** | 1.9±2.3 | 3.4±12.7 | 0.4 |

^*^p-value was calculated by t-tests.

EP, equol producer; NP, non-producer; SI, soy isoflavones; Dai, daidzein; Gen, genistein; Gly, glycitein.
